# Special Issue “Adenovirus Pathogenesis”

**DOI:** 10.3390/v13061112

**Published:** 2021-06-10

**Authors:** Niklas Arnberg, Annasara Lenman

**Affiliations:** 1 Section of Virology, Department of Clinical Microbiology, Umeå University, SE-90185 Umeå, Sweden; 2 Institute for Experimental Virology, TWINCORE, Centre for Experimental and Clinical Infection Research, 30625 Hannover, Germany

Adenovirus is a common cause of disease in humans and in animals. In humans, adenoviruses target different cell types, tissues, and organs and the infection can be either asymptomatic, mild, severe, or lethal. Some infections are acute, whereas others are persistent. Despite being known for almost 70 years, we do not have a full picture of the molecular and cellular mechanisms that determine the outcome of adenovirus infections. This Special Issue contains five original studies and eight reviews that contribute to the overall knowledge of adenovirus pathogenesis.

From the original studies we learn about the contribution of house dust mites to adenovirus infection in advanced in vitro model systems, and the potential impact of adenovirus infection on exacerbations of underlying diseases such as asthma [1]. We dive into the molecular interactions between host cell desmoglein-2 receptors and capsid proteins of a re-emerging adenovirus that is a common cause of acute respiratory distress syndromes: HAdV-B7 [2]. Certain genotypes of a closely related type, HAdV-B14, cause more severe infections than other genotypes within the same serotype. Here, we learn about distinct inflammatory events caused by different genotypes in the Syrian hamster model [3], which have the potential to be useful for dissection of adenovirus genes associated with mild or severe illness. Adenovirus-induced glycolysis is known to be regulated by the protein encoded by the E4orf1 gene. In this Special Issue it is revealed that the 13S isoform (but not the 12S isoform) of the E1A protein also upregulates glycolysis [4]. Finally, we learn that a structural protein, PX, induces apoptosis in hepatocytes, which is associated with a severe illness: hydropericardium hepatitis syndrome in birds [5].

The reviews update us on the molecular and cellular events taking place during more or less severe, persistent infections in eyes [6] and intestine [7]—and a case report describing the death of a neonate suffering from adenovirus-associated sepsis [8]—which remind us about the huge medical need for anti-adenovirus drugs. The Special Issue provides us with up-to-date knowledge about the intracellular functions of cellular RNA-binding zinc finger proteins [9], the DNA damage response [10], viral early proteins [10], and small non-coding RNA [11] during adenovirus replication, as well as the interactions between virions and extracellular proteins and how this contributes to the activation of mononuclear phagocytes [12]. Finally, we are also updated about the physiological roles of the adenovirus dodecahedrons and their potential use in therapeutic development [13].

With this, we would like to acknowledge all authors for their contributions to this Special Issue. It has been a pleasure to read and learn. Together, we will continue our research with the aim to discover and understand known, emerging, and re-emerging adenoviruses, and the molecules and mechanisms that determine tropism, infection, transmission, and pathogenesis. Questions that needs to be addressed include the in vivo role of CAR as the cellular receptor for the fiber capsid protein, and the enigma that neutralizing antibodies target the hexon and (or rather) the fiber protein. Here, further development of animal models will be useful. Altogether, this will enable the development of antiviral drugs, but also more selection and development of more efficient, and safe, adenovirus-based vectors for clinical applications.
